# Exposure to per- and polyfluoroalkyl substances and adult cardiometabolic health: a Canadian Health Measures Survey mixture analysis

**DOI:** 10.1186/s12940-026-01271-1

**Published:** 2026-02-11

**Authors:** Janice M. Y. Hu, Michael M. Borghese, Annie St-Amand

**Affiliations:** Environmental Health Science and Research Bureau, Health Canada, 251 Sir Frederick Banting Driveway, Ottawa, ON K1A 0K9 Canada

**Keywords:** PFAS mixture, Quantile g-computation, Metabolic syndrome, Cardiometabolic risk, Glycated hemoglobin (HbA1c), Glucose metabolism, Perfluorononanoic acid (PFNA)

## Abstract

**Background:**

Metabolic syndrome (MetS) is characterized by four cardiometabolic dimensions including central obesity, dyslipidemia, hypertension, and hyperglycemia, which collectively increase the risk of developing cardiovascular disease and type 2 diabetes mellitus. Epidemiological studies suggest that exposure to per- and polyfluoroalkyl substances (PFAS), which are a class of persistent chemicals used for their water and oil repelling properties, may also contribute to poor cardiometabolic health. We examined associations between exposure to mixture of PFAS and cardiometabolic health among a nationally representative sample of adults living in Canada.

**Methods:**

We used cross-sectional data from 1071 adults aged 20 to 79 from the Canadian Health Measures Survey cycles 2, 5 and 6 (2009–2011, 2016–2019). We examined plasma concentrations of perfluorooctanoic acid (PFOA), perfluorooctane sulfonate (PFOS), perfluorohexane sulfonate (PFHxS), perfluorononanoic acid (PFNA), and perfluorodecanoic acid (PFDA). We examined MetS, a derived cardiometabolic risk score (CMRS), and their individual cardiometabolic components. We used quantile g-computation (qgcomp) to examine joint associations between the PFAS mixture and cardiometabolic outcomes, and qgcomp weights to determine individual PFAS contributions to the overall mixture effect. Furthermore, we used modified Poisson and linear regression to estimate associations for individual PFAS plasma concentrations and compare with our qgcomp results. Analyses were stratified by sex.

**Results:**

The prevalence of MetS is 27% in adults living in Canada. In qgcomp models, we observed null associations between the PFAS mixture and both MetS (prevalence ratio: 0.90; 95% CI: 0.74, 1.19) and CMRS (regression index: -0.10; 95% CI: -0.32, 0.12). Each one-quartile increase in the PFAS mixture was associated with 1.2% higher glycated hemoglobin (HbA1c) levels among total population and 1.6% among females, with PFNA contributing most to the joint associations. The PFAS mixture showed null associations with other MetS components. Results from our linear regression models corroborated the findings from the mixture analysis with directions consistent with the qgcomp effect estimates and weights.

**Conclusion:**

Using cross-sectional data from a nationally representative sample of adults living in Canada, our findings suggest that a mixture of PFAS may adversely affect glucose metabolism. Further prospective studies are needed to corroborate these findings and establish temporality.

**Supplementary Information:**

The online version contains supplementary material available at 10.1186/s12940-026-01271-1.

## Background

Metabolic syndrome (MetS) is a clinical diagnosis defined according to a cluster of cardiometabolic risk factors (CMRFs) that can be categorized into four dimensions: central obesity, dyslipidemia, hypertension, and hyperglycemia. MetS and the associated CMRFs are important contributors to the development of cardiovascular diseases (CVD) and type 2 diabetes mellitus (T2D), the two leading causes of death worldwide [1, 2]. In 2018, the estimated global prevalence of MetS among adults was 25% [3]. In Canada, the estimated prevalence was 26% between 2016 and 2019 [4]. Cumulatively, over one billion people had MetS [3, 4]. As a result, identifying and addressing the cluster of modifiable risk factors related to MetS (e.g., elevated waist circumference (WC), triglycerides (TG), blood pressure (BP) and blood glucose (GLU) levels) may help decrease the incidence of T2D and CVDs and lower the overall disease burden. Exposure to per- and polyfluoroalkyl substances (PFAS) may also be one such modifiable risk factor.

PFAS are a class of persistent chemicals used in everyday products for their water and oil repelling properties [5]. Mechanisms underlying PFAS toxicity in humans are not fully understood, but PFAS have been shown to adversely affect lipid and glucose metabolism via oxidative stress, inflammation, metabolic hormones, or even sex steroid hormones [6, 7]. As a result, the motive to investigate PFAS in relation to cardiometabolic health risks has increased.

Epidemiological studies have demonstrated that exposure to PFAS is deleteriously associated with individual cardiometabolic risk factors, including elevated BP and lipid levels (i.e., higher total cholesterol and low-density lipoprotein cholesterol (LDL-C)) [8, 9]. However, evidence for associations between exposure to PFAS and central adiposity, glucose metabolism, and MetS remains inconsistent [8–14]. Therefore, further studies to examine the associations with MetS and cardiometabolic risk factors are needed [11]. Moreover, we are exposed to multiple PFAS concurrently but available studies have mostly examined associations with PFAS individually. Therefore, there is a need to consider the potential cumulative effects of a mixture of PFAS on MetS and its components. Finally, few studies have conducted sex-stratified analyses [11], which is essential given the known sex differences in both PFAS toxicokinetics (i.e., as a result of sex-specific excretion routes such as pregnancy, breastfeeding, and menstruation [5]), as well as, MetS [15] and its components [16].

To address these knowledge gaps, we investigated the potential associations between exposure to PFAS and outcomes related to cardiometabolic health, both overall and stratified by sex, among a large population of adults recruited as part of the Canadian Health Measures Survey (CHMS) cycles 2, 5 and 6 (2009–2011, 2016–2019). Specifically, our main objective was to conduct quantile g-computation (qgcomp) analysis to evaluate the associations between a mixture of plasma PFAS concentrations and (1) MetS diagnosis, a binary composite indicator of cardiometabolic risk based on clinical risk factors, (2) cardiometabolic risk score (CMRS), a continuous composite indicator of MetS severity score, and (3) individual components of CMRS and MetS (i.e., WC, TG, GLU, high-density lipoprotein cholesterol (HDL-C), systolic (SBP) and diastolic BP (DBP), and glycated hemoglobin (HbA1c)). We also examined PFAS concentrations individually using Poisson and linear regressions to compare with our qgcomp mixture results.

## Methods

### Study design and population

We used data from cycles 2 (2009–2011), 5 (2016–2017) and 6 (2018–2019) of the CHMS, a nationally representative and repeated cross-sectional survey that collects Canadian health-related information through household interviews, questionnaires and direct physical measurements. Detailed information on the sampling methodology has been published [17, 18]. Briefly, a stratified multistage sampling strategy was used to select participants across 18 sites in cycle 2 and 16 sites in both cycles 5 and 6 from 5 Canadian regions. The CHMS is representative of 96% of the Canadian population and excludes those living on reserves and other Indigenous settlements, institutionalized populations, full-time members of the Canadian forces, and residents of certain remote regions. Participants completed a household interview and were subsequently invited to visit a mobile examination centre where physical health measurements and blood samples were collected. The CHMS was approved by the Health Canada and Public Health Agency of Canada Research Ethics Board and written informed consent/assent was obtained from all participants.

In this analysis, we included 1,071 individuals aged 20 to 79 years who provided blood samples, demographic, and clinical data. We excluded participants who reported being pregnant (*n* = 22) and had type 1 diabetes (*n* = 25).

### Measurement of plasma PFAS

Plasma concentrations of nine PFAS were analyzed during CHMS cycles 2, 5 and 6. Biospecimen collection was carried out in mobile examination centres and detailed descriptions have been published [19–21]. PFAS detected in at least 70% of samples were retained in the current analysis and the measurements below the limit of detection (< LOD) were replaced using the single imputation “fill-in” lognormal distribution with mean and standard deviation (SD) estimated from the observed data [22]. This approach yields unbiased regression coefficient estimates if the imputation distribution is correct [22]. The PFAS retained were perfluorooctanoic acid (PFOA), perfluorooctane sulfonate (PFOS), perfluorohexane sulfonate (PFHxS), perfluorodecanoic acid (PFDA) and perfluorononanoic acid (PFNA). Due to shared toxicokinetic properties, similar accumulation and long half-lives, we included all 5 PFAS in our mixture and also summed the concentrations of all five PFAS to obtain total PFAS (∑ 5PFAS), a composite measure, for use in our modified Poisson and linear regression models. Furthermore, to reduce the potential influence of outliers due to the right skewed distributions of PFAS concentrations and to obtain a more linear dose-response relationship for improved model fit, each PFAS and total PFAS concentrations were log_2_-transformed before inclusion in the statistical models. The transformed unit allows the regression coefficients to be interpreted as each two-fold increase in plasma concentration.

### Outcome measures

MetS is a clinical diagnosis defined according to a cluster of CMRFs that can be categorized into four dimensions: central obesity, dyslipidemia, hypertension, and hyperglycemia. Individuals are diagnosed with MetS if they meet three or more of the following clinical criteria [23]:

- Waist circumference (WC) ≥ 102 cm for males and ≥ 88 cm for females or for those who reported Asian ethnicity ≥ 90 cm for males and ≥ 80 cm for females.
- Elevated triglycerides (TG) ≥ 150 mg/dL (1.7 mmol/L) or reported taking medications for elevated TG.
- Reduced high-density lipoprotein cholesterol (HDL-C) < 40 mg/dL (1.0 mmol/L) for males and < 50 mg/dL (1.3 mmol/L) for females or reported taking medications for reduced HDL-C.
- Elevated blood pressures (BPs) systolic ≥ 130 and/or diastolic ≥ 85 mmHg or reported taking antihypertensive medication for reducing blood pressure, or were previously diagnosed with hypertension.
- Elevated glycated hemoglobin (HbA1c) ≥ 5.7% (39 mmol/L) or reported taking medication for elevated glucose, or were previously diagnosed with type 2 diabetes mellitus (T2D).

We examined MetS as a binary variable. MetS typically includes elevated fasting blood glucose measures but glucose measurements from CHMS cycles 2, 5, and 6 were from non-fasting samples, so we used instead the equivalent HbA1c levels according to the American Diabetes Association [24]. Moreover, HbA1c is a reliable clinical indicator of elevated blood glucose concentrations over the previous 2–3 months that can be substituted for fasting plasma glucose concentrations [25, 26]. Furthermore, as Canada is a multicultural country, for participants who reported Asian ethnicity (i.e., South Asian, Chinese, Filipino, Southeast Asian, Korean, or Japanese), a lower WC threshold of 90 cm for males and 80 cm for females were used [23].

The approach of considering MetS as a binary variable has drawbacks, such as overlooking individuals with borderline abnormalities who are classified negative for MetS and considered low-risk, despite having MetS components that are measured just below the clinical cut-off values. Additionally, the risk associated with each component of MetS has an underlying continuous distribution rather than dichotomous. Therefore, using a continuous MetS score can circumvent the limitations of dichotomization, allow us to evaluate the degree of severity of MetS, and provide increased sensitivity and statistical power. As a result, in addition to the binary classification of MetS, we also examined CMRS, a continuous MetS severity score of cardiometabolic health risk.

The CMRS was constructed based on population-specific Z-scores of five cardiometabolic risk factors (CMRFs): WC, whole blood HbA1c, serum TG, serum HDL-C, and mean arteriole pressure (MAP) calculated using SBP and DBP [27]. For each CMRF, we used an internal standardization approach by calculating Z-scores using age- and sex-standardized residuals from a linear regression model with the CMRF as dependent variable and age and sex as independent variables. We calculated the MAP Z-score (zMAP) as zDBP + [⅓(zSBP - zDBP)], where zDBP and zSBP are Z-scores of DBP and SBP, respectively. To generate a value indicative of an individual’s average CMRS, we summed all Z-scores, except for HDL-C, which was subtracted from the overall CMRS to account for its protective effect on cardiometabolic health. Subsequently, to preserve Z-score distribution [28], the sum of Z-scores were divided by the square root of the number of CMRF:

$$\begin{aligned}&\text {mean}\mathrm{CMRS}=\frac{zWC\:+\:zHbA1c\:+\:zTG\:-\:zHDL\:+\:zMAP}{\surd\:nCMRF}\end{aligned}$$

where zWC, zHbA1c, zTG, zHDL and zMAP are the Z-scores of CMRFs and nCMRF represents the number of risk factors included in the formula. The mean CMRS indicates, on average, whether an individual’s CMRFs are beyond the healthy range and higher scores correspond to a less favourable cardiometabolic risk profile.

We also examined individual components of CMRS and MetS to better understand any association observed for the overall CMRS. WC was measured in centimeters (cm) using the National Institute of Health protocol [29]. BP measurements were taken at rest using an oscillometric BP measurement device where six measurements were taken at one minute intervals following a five minute rest period. The last five measurements were used to calculate the average resting BPs measured in millimeters of mercury (mmHg) [30]. TG was analyzed in serum samples of individuals who had fasted for a minimum of 10 h and reported in millimole per litre (mmol/L), respectively. HDL and HbA1c were analyzed in blood samples of non-fasting individuals. HDL was measured in serum (mmol/L) and HbA1c was measured in whole blood and expressed as a percentage (%). In addition to the CMRFs noted above, we also included non-fasting serum glucose (mmol/L).

We log_2_-transformed CMRFs to address normality of the residual and reduce the potential influence of outliers before inclusion in the models. The results (β) were subsequently back-transformed using the formula (2^β^-1)*100 and interpreted as the percent difference (%Δ) in each specific CMRF.

### Covariates

We selected covariates using a directed acyclic graph (Supplemental Figure S1) by considering factors associated with both exposure to PFAS and cardiometabolic outcomes. All models were adjusted for age (continuous), sex (male; female), race/ethnicity (white; non-white), highest level of education (elementary school graduates or less; secondary school graduates; post-secondary school graduate), cigarette smoking status (never; ever), marital status (single; married or common-law; widowed, separated or divorced), country of birth (Canada; foreign), average daily moderate to vigorous physical activity (continuous; minutes/day), fish and shellfish consumption (continuous; times/month), and survey cycle (cycle 2, 5 and 6). For models including females only, we additionally adjusted for parity (nulliparous; had one live birth; had two live births; had three or more live births). For CMRS models, we did not adjust for age and sex because these were standardized for when creating the Z-scores.

### Statistical analysis

#### Descriptive analysis

We examined the participant characteristics and various cardiometabolic health indicators by calculating weighted and unweighted sample sizes (n), proportions (%), means, and standard deviations (SD). Questionnaire responses reported as “Not stated”, “Refusal”, or “Don’t know” were treated as missing data. For PFAS, we present weighted geometric means (GM) and percentiles as well as pairwise Spearman’s correlation coefficients of PFAS concentrations. We stratified all analyses by sex. We observed fewer than 1% missingness in all sociodemographic characteristics and cardiometabolic health indicators except for physical activity at 25%. This is because physical activity monitors were only offered to a subsample of participants. We carried out complete case analyses with final analytical sample sizes of 1071 for total population, 513 (48%) for males and 558 (52%) for females. All analyses were conducted using R Statistical Software version 4.3.2 [31].

#### PFAS mixture analysis: quantile g-computation (qgcomp)

We examined the joint associations between exposure to a mixture of five PFAS and cardiometabolic outcomes of interest (i.e., MetS, CMRS and CMRFs) using quantile g-computation (qgcomp) [32]. Qgcomp estimates the overall mixture effect by first transforming all chemical concentrations into quantiles, then fitting a linear model by regressing the outcome onto the set of quantized exposures and covariates. Qgcomp provides an estimate of the overall joint effect (Ѱ) along with weights that can be interpreted as adjusted, independent, and scaled effect sizes for quantized exposures, which reflect the relative contribution of each specific PFAS to the overall joint effect. Weights in the same direction sum to 1 and can be compared relative to the weights in the same direction. For MetS, we fit Poisson models and exponentiated Ѱ to estimate the prevalence ratio (PR) which can be interpreted as the difference in probability of having MetS that is associated with a one quartile increase in all PFAS exposures within the mixture. For CMRS and CMRFs, we fit Gaussian models and interpreted Ѱ as the difference in mean CMRS, or back-transformed Ѱ to determine the percent difference (%Δ) in each CMRF associated with a one quartile increase in all PFAS exposures within the mixture. Furthermore, given the complex survey design and sampling methodology of the CHMS, we incorporated bootstrapped weights into all models to obtain valid estimates of variance (95% CI) and allow for the interpretation of results as being nationally representative. Qgcomp was carried out using the qgcomp package [33]. Weighted regression analysis was carried out using the survey package [34].

#### Individual PFAS analysis: survey-weighted modified Poisson and linear regressions

To complement and examine the robustness of the mixture analysis results, we examined associations between individual PFAS concentrations and MetS, CMRS, and CMRFs. For MetS, we conducted survey-weighted modified Poisson regression with a robust error variance [35] to estimate PRs. Resulting PR indicates the difference in probability of having MetS with each 2-fold increase in PFAS concentrations. Furthermore, for continuous outcomes (i.e., CMRS and CMRFs), we conducted survey-weighted linear regression analyses. The results describe the difference in mean CMRS and the percent difference in mean CMRF associated with each 2-fold increase in PFAS concentrations.

#### Effect modification and sensitivity analyses

We conducted stratified analyses to examine the potential modifying effect of sex on the relationship between PFAS concentrations and all outcomes of interest. For stratified models, we did not adjust for sex. We also conducted sensitivity analyses for continuous CMRS and CMRFs where we excluded some individuals who reported taking medications. For analysis of cholesterol outcomes (i.e., TG and HDL-C), we excluded individuals taking lipid-lowering medications (*n* = 306; 20%). For the analysis of blood pressures (i.e., DBP and SBP), we excluded individuals taking antihypertensive medications (*n* = 320; 21%). For the analysis of glucose metabolism (i.e., GLU and HbA1c), we excluded individuals taking diabetes medications (*n* = 102, 7%).

## Results

### Descriptive statistics

We included 1071 CHMS participants from cycles 2, 5 and 6 and presented the unweighted sample size (n) and weighted proportions (%) for all individuals and stratified by sex (Table 1). The CHMS participants were on average 50 years old, predominantly white (77%), had a post-secondary degree (69%), married or common-law (66%), never smoked (81%), and were born in Canada (70%). They, on average, consumed fish and shellfish 8 times (standard deviation (SD) = 9) per month and engaged in 23 min (SD = 24) of moderate to vigorous physical activity per day. The average levels of all CMRFs were within normal ranges. Overall, 27.1% of adults living in Canada had MetS (25.5% for males and 28.5% for females); between cycles 2 and 6 (2009–2019), the prevalence increased from 24.0% to 33.7%, rising from 27.9% to 28.2% among males and from 20.4% to 37.5% among females.

**Table 1:** Sociodemographic characteristics and health indicators for participants in cycles 2, 5, and 6 of the Canadian health measures survey (2009–2011, 2016–2019)

	Total (*n* = 1071)	Males (*n* = 513)	Females (*n* = 558)
Demographic characteristics (*n* (weighted %) or mean (weighted SD)			
Cycle			
2	398 (30.9)	191 (32.5)	207 (29.5)
5	352 (36.7)	171 (38.9)	180 (34.8)
6	321 (32.4)	150 (28.6)	171 (35.7)
Age (years)	49.8 (15.5)	49.5 (15.7)	50.0 (15.6)
Race/Ethnicity			
White	854 (76.5)	399 (77.1)	611 (76.0)
Other	217 (23.5)	114 (22.9)	150 (24.0)
Education			
High school or less	89 (5.6)	50 (6.1)	39 (5.1)
Secondary school	215 (25.3)	95 (25.5)	120 (25.0)
Post-secondary graduation	767 (69.2)	368 (68.4)	399 (69.9)
Marital status			
Married or common-law	755 (65.7)	396 (67.4)	359 (64.3)
Widowed, separated, or divorced	162 (9.0)	48 (6.2)	114 (11.3)
Single	154 (25.3)	69 (26.4)	85 (24.4)
Smoking status			
Never	900 (80.8)	418 (72.9)	482 (87.6)
Ever	171 (19.2)	95 (27.1)	76 (12.4)
Country of birth			
Canadian-born	780 (69.9)	359 (71.2)	421 (68.9)
Foreign-born	291 (30.1)	154 (28.8)	173 (31.1)
Parity			
0	-	-	117 (32.2)
1	-	-	100 (11.5)
2	-	-	222 (38.8)
3+	-	-	113 (17.5)
Fish and shellfish consumption (number of times/month)	7.67 (8.80)	7.31 (8.70)	8.00 (8.84)
Average daily moderate to vigorous physical activity (mins/day)	22.7 (24.1)	25.1 (26.3)	20.4 (21.9)
Cardiometabolic health indicators (n (weighted %) or mean (weighted SD))			
Metabolic syndrome (MetS)			
Yes	328 (27.1)	157 (25.5)	171 (28.5)
No	743 (72.9)	356 (74.5)	387 (71.5)
Cardiometabolic risk score (CMRS)	-0.07 (1.30)	-0.06 (1.20)	-0.09 (1.39)
Non-fasting serum glucose (GLU) (mmol/L)	5.41 (1.21)	5.58 (1.08)	5.25 (1.29)
Waist circumference (WC) (cm)	93.5 (15.4)	96.7 (14.0)	90.6 (16.3)
Fasting serum triglycerides (TG) (mmol/L)	1.35 (0.70)	1.48 (0.76)	1.23 (0.65)
Serum high density lipoprotein cholesterol (HDL-C) (mmol/L)	1.44 (0.42)	1.29 (0.36)	1.58 (0.43)
Average resting systolic blood pressure (SBP) (mmHg)	114 (16)	115 (14)	113 (17)
Average resting diastolic blood pressure (DBP) (mmHg)	72 (10)	74 (9)	70 (11)
Whole blood glycated hemoglobin A1c (HbA1c) (%)	5.57 (1.30)	5.62 (0.67)	5.53 (0.73)

Unweighted sample size (n) and means are presented alongside weighted proportions (%) and weighted standard deviations (SD)

Males and females had mostly similar characteristics except that on average, females tended to smoke less (12 vs. 27%), eat fish and shellfish more frequently (8 vs. 7 times/month), and have higher HDL-C measurement (1.6 vs. 1.3 mmol/L), compared to males. Females were also less physically active (20 vs. 25 min/day) and had lower WC (91 vs. 97 cm), TG (1.2 vs. 1.5 mmol/L), GLU (5.3 vs. 5.6 mmol/L) and DBP (70 vs. 74 mmHg) levels compared to males. Furthermore, 32% of females were nulliparous and 50% had one or two live births.

Most PFAS were detected in > 95% of participants, except for PFDA which was detected in 82% of participants (84% for males and 80% for females (Supplemental Table S1)). Males had higher GM plasma PFAS concentrations compared to females, especially for PFOS (5.2 vs. 3.2 ug/L) and PFHxS (1.6 vs. 0.8 ug/L). We observed mostly moderate to strong correlations between individual PFAS with Spearman’s correlation coefficients ranging from 0.3 to 0.8 for both total sample and when stratified by sex (Supplemental Figure S2).

### PFAS mixture analysis using Qgcomp

In our adjusted qgcomp models, joint associations between the mixture of five PFAS and MetS were null, with 95% CIs including 1.0. The association was in the negative direction for total population (PR = 0.90; 95% CI: 0.74, 1.19). The PRs for sex-stratified analyses were aligned with the null value (PR_males_ = 1.08; 95% CI: 0.76, 1.48 and PR_females_ = 1.00; 95% CI: 0.69, 1.40) (Table 2).

**Table 2:** Prevalence ratios (PR) and 95% confidence intervals (95% CI) and weights for associations between a one-quartile increase in the PFAS mixture and metabolic syndrome (MetS) among participants aged 20–79 in cycles 2, 5, and 6 of the Canadian health measures survey (2009–2011, 2016–2019), using survey-weighted quantile g-computation regression

	PR (95% CI)	Weights					
		Direction	PFOA	PFOS	PFHxS	PFDA	PFNA
Total	0.90 (0.74, 1.19)	Positive		0.12			0.88
		Negative	0.25		0.24	0.50	
Males	1.08 (0.76, 1.48)	Positive	0.37	0.37	0.26		0.00
		Negative				1.00	
Females	1.00 (0.69, 1.40)	Positive		0.51			0.49
		Negative	0.30		0.16	0.54	

All models are adjusted for age, race/ethnicity, education, marital status, smoking, country of birth, shellfish/fish consumption, physical activity and survey cycle. Model including total population was additionally adjusted for sex and model including females only was additionally adjusted for parity. Positive and negative weights correspond to the proportion of the effect in a particular direction and each sums to 1

In the adjusted qgcomp models estimating the overall mixture effect of PFAS on CMRS, we also observed null results with 95% CI including 0 (Table 3). In the total population model, the joint association was in the negative direction (Ѱ_total_ = -0.10; 95% CI: -0.32, 0.12). In sex-stratified analyses, the joint association was also in the negative direction for females (Ѱ_females_ = -0.21; 95% CI: -0.48, 0.15), and in males, the overall joint effect for CMRS aligned with the null value (Ѱ_males_ = 0.02; 95% CI: -0.28, 0.29). For both MetS and CMRS, qgcomp weights identified PFDA as the main contributor to the joint associations in total and female populations.

**Table 3:** Overall joint effect (Ψ) and 95% confidence intervals (95% CI) and weights for associations between a one-quartile increase in the PFAS mixture and cardiometabolic risk score (CMRS) among participants aged 20–79 in cycles 2, 5, and 6 of the Canadian health measures survey (2009–2011, 2016–2019), using survey-weighted quantile g-computation regression

	Ψ (95% CI)	Weights					
		Direction	PFOA	PFOS	PFHxS	PFDA	PFNA
Total	-0.10 (-0.32, 0.12)	Positive	0.20				0.80
		Negative		0.34	0.27	0.39	
Males	0.02 (-0.28, 0.29)	Positive	0.53				0.47
		Negative		0.29	0.35	0.36	
Females	-0.21 (-0.48, 0.15)	Positive					1.00
		Negative	0.27	0.06	0.20	0.48	

All models are adjusted for race/ethnicity, education, marital status, smoking, country of birth, shellfish/fish consumption, physical activity and survey cycle. Model including females only was additionally adjusted for parity. Positive and negative weights correspond to the proportion of the effect in a particular direction and each sums to 1

In our adjusted qgcomp models for CMRFs, results were largely null except for HbA1c (Table 4). Each one-quartile increase in the mixture of five PFAS was associated with 1.2% (95% CI: 0.1, 2.0) higher HbA1c among the total population. Among females, although the 95% CIs included the null, an increase in HbA1c of 1.6% (95% CI: -0.1, 3.0) was observed, while a weaker association was observed among males with 0.7% (95% CI: -0.7, 1.8). PFNA, with the largest positive weights, contributed most to the joint associations with HbA1c.

**Table 4:** Percent differences (%Δ) and 95% confidence intervals and weights for associations between a one-quartile increase in the PFAS mixture and cardiometabolic risk factors (CMRFs) among participants aged 20–79 in cycles 2, 5, and 6 of the Canadian health measures survey (2009–2011, 2016–2019), using survey-weighted quantile g-computation regression

		%Δ (95% CI)	Weights					
			Direction	PFOA	PFOS	PFHxS	PFDA	PFNA
Waist circumference (WC)	Total	-1.57 (-4.02, 0.25)	Positive	0.06				0.94
			Negative		0.03	0.53	0.44	
	Males	0.09 (-3.32, 2.35)	Positive	0.70				0.30
			Negative		0.03	0.60	0.38	
	Females	-3.36 (-6.48, 0.97)	Positive		0.59			0.41
			Negative	0.10		0.47	0.43	
Glucose (GLU)	Total	1.50 (-0.41, 2.91)	Positive	0.30		0.19	0.51	
			Negative		0.58			0.43
	Males	1.27 (-1.23, 2.76)	Positive	0.72		0.27	0.02	
			Negative		0.75			0.25
	Females	1.73 (-0.80, 3.40)	Positive			0.25	0.75	0.00
			Negative	0.04	0.96			
Triglycerides (TG)	Total	4.89 (-7.12, 16.07)	Positive			0.04		0.96
			Negative	0.13	0.31		0.56	
	Males	-1.60 (-11.12, 12.75)	Positive	0.70		0.01		0.29
			Negative		0.38		0.62	
	Females	-0.39 (-11.81, 14.67)	Positive	0.08	0.26			0.66
			Negative			0.65	0.35	
Systolic blood pressure (SBP)	Total	0.10 (-2.66, 2.78)	Positive	0.74			0.26	
			Negative		0.56	0.04		0.41
	Males	1.34 (-1.92, 3.27)	Positive	0.49				0.51
			Negative		0.07	0.68	0.25	
	Females	-0.93 (-3.52, 2.48)	Positive			0.55	0.45	
			Negative	0.38	0.41			0.21
Diastolic blood pressure (DBP)	Total	-1.21 (-4.37, 2.16)	Positive	0.66			0.34	
			Negative		0.43	0.23		0.35
	Males	1.92 (-1.54, 3.79)	Positive	0.63	0.04		0.22	0.11
			Negative			1.00		
	Females	-2.84 (-5.65, 1.91)	Positive			0.68	0.32	
			Negative	0.35	0.59			0.07
High-density lipoprotein cholesterol (HDL-C)	Total	2.45 (-1.12, 6.47)	Positive	0.10	0.27	0.15	0.48	
			Negative					1.00
	Males	4.86 (-1.28, 10.71)	Positive		0.35	0.28	0.37	
			Negative	0.29				0.71
	Females	3.93 (-2.12, 9.18)	Positive	0.22		0.25	0.53	
			Negative		0.39			0.61
Glycated hemoglobin (HbA1c)	Total	1.16 (0.10, 1.97)**	Positive	0.02	0.39			0.59
			Negative			0.11	0.89	
	Males	0.72 (-0.66, 1.76)	Positive	0.36	0.18	0.35		0.12
			Negative				1.00	
	Females	1.58 (-0.08, 2.98)*	Positive		0.17			0.84
			Negative	0.39		0.08	0.53	

All models are adjusted for age, race/ethnicity, education, marital status, smoking, country of birth, shellfish/fish consumption, physical activity and survey cycle. Models including total population were additionally adjusted for sex and models including females only were additionally adjusted for parity. Positive and negative weights correspond to the proportion of the effect in a particular direction and each sums to 1

^**^p-value < 0.05, ^*^p-value < 0.10. TG and HDL models excluded individuals on lipid-lowering medications; GLU and HbA1c models excluded individuals taking diabetes medications; SBP and DBP models excluded individuals on antihypertensive medications

### Individual PFAS analysis using modified Poisson and linear regressions

**Fig. 1:**
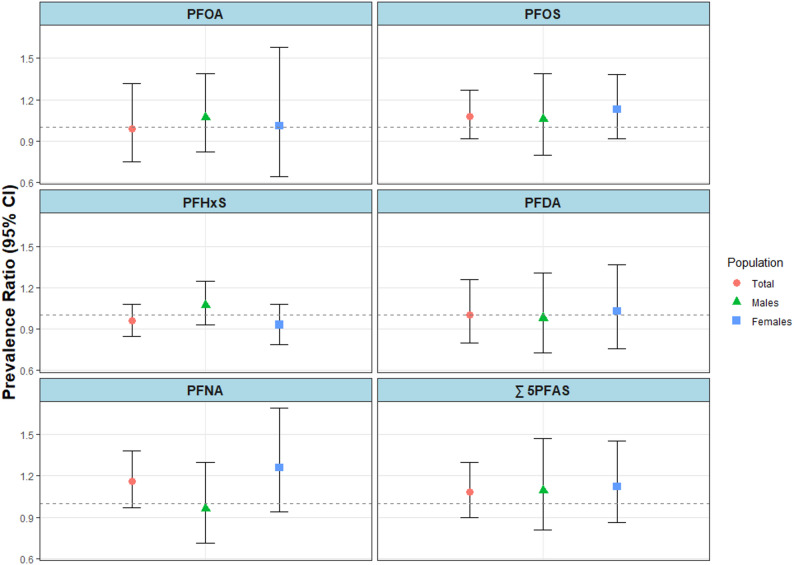
Adjusted prevalence ratios (95% CIs) for associations between each 2-fold increase in PFAS plasma concentrations and metabolic syndrome (MetS) among participants aged 20–79 in cycles 2, 5, and 6 of the Canadian Health Measures Survey (2009–2011, 2016–2019), using survey-weighted modified Poisson regression

**Fig. 2:**
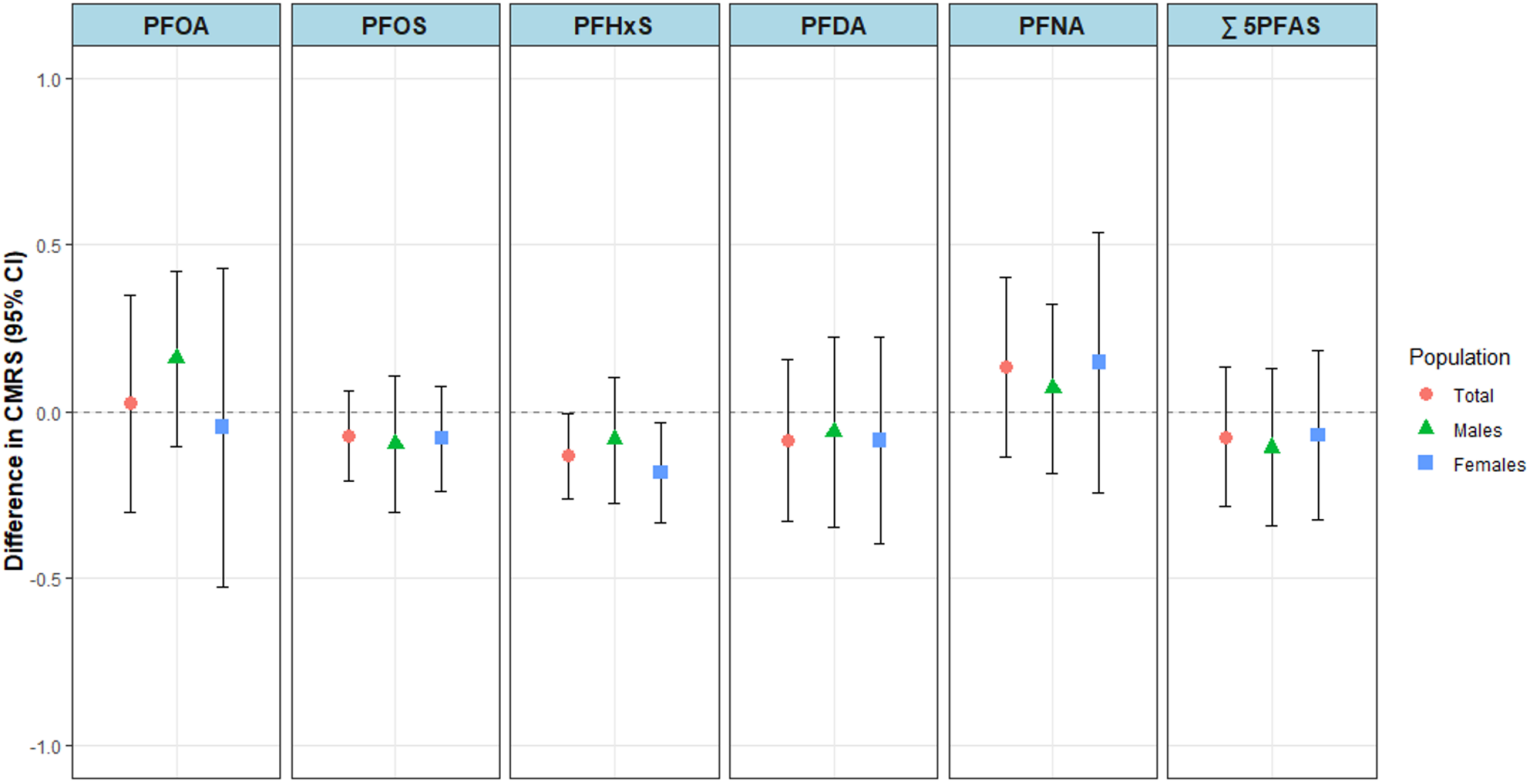
Adjusted mean differences (95% CI) for associations between each 2-fold increase in plasma PFAS concentrations and cardiometabolic risk score (CMRS) among participants aged 20–79 cycles 2, 5, and 6 of the Canadian Health Measures Survey (2009–2011, 2016–2019), using survey-weighted linear regression

We estimated individual associations in adjusted modified Poisson regression models for the association between exposure to individual PFAS and MetS (Fig. 1 and Supplemental Table S2). In the adjusted models, we observed null associations with PRs ranging from 0.96 (95% CI: 0.85, 1.08) to 1.16 (95% CI: 0.97, 1.38) among total participants, 0.96 (95% CI: 0.71, 1.30) to 1.09 (95% CI: 0.81, 1.47) among males, and 0.93 (95% CI: 0.79, 1.08) to 1.26 (95% CI: 0.94, 1.69) among females. In our adjusted linear regression models estimating the difference in CMRS associated with individual PFAS, we also observed all null associations except for the associations with PFHxS among the total population (Δ = -0.13; 95% CI: -0.26, -0.00) and among females (Δ = -0.18; 95% CI: -0.33, -0.03) (Fig. 2 and Supplemental Table S3).

**Fig. 3:**
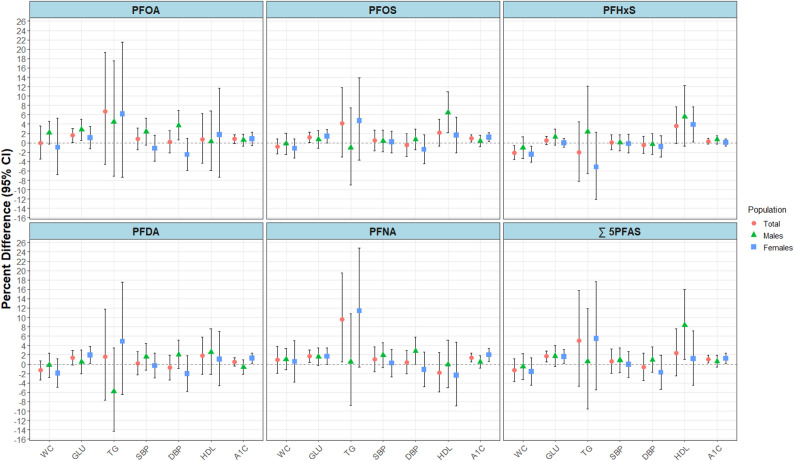
Adjusted percent differences (95% CI) for associations between each 2-fold increase in plasma PFAS concentrations and cardiometabolic risk factors (CMRFs) among participants aged 20–79 cycles 2, 5, and 6 of the Canadian Health Measures Survey (2009–2011, 2016–2019), using survey-weighted linear regression

In the adjusted linear regression models for CMRFs, we observed associations with HbA1c that were similar to those in qgcomp mixture models (Fig. 3 and Supplemental Table S4). Each 2-fold increase in individual PFOS, PFNA and ∑ 5PFAS was associated with up to 1.4% (95% CI: 0.5, 2.4) higher HbA1c values among total participants. For females, each 2-fold increase in PFOS, PFDA, PFNA, and ∑ 5PFAS was associated with up to 2.0% (95% CI: 0.6, 3.3) higher HbA1c levels. Similar associations were observed for fasting GLU, with 2-fold increases in PFOA, PFOS, PFNA, and ∑ 5PFAS associated with up to 1.7% higher values (95%CI: 0.4, 3.0) in the total population; this was mostly driven by stronger associations observed among females, with the exception of PFOA among males. Furthermore, each 2-fold increase in PFHxS was associated with 2.5% (95% CI: -4.2, -0.7) lower WC and 3.9% (95% CI: 0.2, 7.7) higher HDL-C levels among females. Lastly, we observed inconsistent associations with TG, SBP and DBP for some PFAS, varying across total and sex-stratified models.

## Discussion

Using repeated, cross-sectional data from a nationally representative sample of adults living in Canada from 2009 to 2011 and 2016 to 2019, we showed that plasma concentrations of most individual PFAS, ∑ 5PFAS, and the mixture of five PFAS were adversely associated with glucose metabolism as measured by HbA1c. We observed null associations with MetS and CMRS. We also observed null associations with other CMRFs. Results from linear regression models generally corroborated the mixture analyses with directions of the association consistent with the qgcomp weights.

### PFAS and hyperglycemia

Reviews of epidemiological studies that have modeled associations between PFAS exposure and impaired glucose metabolism using indicators such as fasting serum glucose, fasting serum insulin and Homeostatic Model Assessment of Insulin Resistance (HOMA-IR) have reported inconsistent results [11, 14, 36] with reports of positive [37–43], negative [44–46] and null associations [47–51]. Few studies have examined HbA1c, which reflects average blood glucose levels over the lifespan of a hemoglobin of approximately 120 days. Among the limited studies, the findings for HbA1c were also inconsistent with some reporting positive associations [52–56], two reporting null associations [54, 57], and one reporting a negative association [41].

Among the seven studies identified, three examined PFAS as a mixture. The first was by Kang and Kim (2024), a comparable study to ours that included the same PFAS mixture components and used national health survey data from South Korea (KoNEHS 2018–2020) with similar age range [54]. In their study, serum PFAS concentrations were at least three times higher than in the current study of adults living in Canada, despite being from a more recent time point. They reported an association between the PFAS mixture and elevated HbA1c using Bayesian kernel machine regression (BKMR) and identified PFOS as the main contributor, whereas PFNA was the main contributor in our Canadian study. Likewise, Chung et al. (2024), who examined the same Korean population and the same mixture components but utilized the mixture risk assessment relative potency factor (Cmix) approach, also reported a positive association with HbA1c [55]. Finally, Han et al. (2021), in their case control study, conducted a qgcomp analysis and observed null associations between HbA1c and exposure to a PFAS mixture consisting of the same five PFAS as ours plus perfluoroundecanoic acid (PFUnDA) and 6:2 chlorinated polyfluoroalkyl ether sulfonic acid (6:2 Cl-PFESA) among a sample of 304 Chinese adults aged 25 to 74 with T2D recruited in 2016–2017 [57]. However, given the ubiquitous exposure to 6:2 Cl-PFESA in China [58], the relevance of this PFAS mixture to that of which adults living in Canada are exposed to is unclear.

Single-pollutant studies from the United States [52, 56], China [53], and Spain [59] suggest that some individual PFAS are deleteriously associated with glucose metabolism. For example, in a prospective study of 957 American adults at high risk for developing T2D recruited between 1996 and 1999, Cardenas et al. (2017) observed positive associations with PFOS and PFOA exposures, but null associations with PFNA [56]. Similarly, Brosset and Ngueta (2023) who examined National Health and Nutrition Examination Survey (NHANES) (1999–2018, *n* = 4575, aged ≥ 65 with T2D) also observed positive association between some PFAS (i.e., PFNA and PFHxS) and elevated HbA1c [52]. They also found, contrary to our results, positive associations for PFDA in males and null associations in females [52]. The sole study that reported negative associations was by Liu et al. (2018) who examined PFOS and PFOA isomers (structurally arranged as either linear or branched forms). They reported that all isomers of PFOA were negatively associated with HbA1c among the 1871 NHANES (2013-14) adult participants examined and concluded that there might be different toxicokinetic or metabolic effects of PFOA isomers in humans [41]. The exact mechanism that explains how PFAS affects HbA1c or glucose metabolism in humans is not fully understood, but exposure to PFNA, the main contributor to the overall joint associations with HbA1c we observed, has been shown to increase serum glucose levels, suppress insulin signal pathway and induce hepatic oxidative stress in rats [60]. There is a need for further investigations to develop a more comprehensive understanding of the effects of PFAS on glucose metabolism.

### PFAS and MetS or CMRS

Epidemiological studies linking MetS and exposure to PFAS are limited with inconclusive results [61]. Based on a meta-analysis of 12 studies published before January 2021, the evidence does not support an association between PFAS (i.e., PFOA, PFOS, PFNA, PFHxS) and MetS [62] but more recent publications showed varying results [55, 63–65]. For example, similarly to our findings, Lin et al. (2025) reported null associations in their age-stratified mixture analyses where they conducted weighted quantile sum (WQS) and BKMR analyses with NHANES (2003–2018) data for 5850 American adults. However, their single-pollutant analysis showed negative associations with MetS for PFHxS and 2-(N-Methyl-perfluorooctane sulfonamido) acetic acid (MeFOSAA) among young (aged 20–39) and middle-aged adults (aged 40–59) [63]. Conversely, in a cross-sectional study that included 2984 adults who took part in KoNEHS Cycle 4 (2018–2020), Chung et al. (2024) observed that the PFAS mixture (Cmix) was associated with higher odds of MetS in females only. Their BKMR analysis revealed PFNA as the main contributor [55]. In sum, the available evidence for potential associations between mixtures of PFAS is heterogenous and inconsistent. Prospective studies are needed to better understand the potential contributions of PFAS, both individually and as a mixture, to MetS. Furthermore, these studies should consider examining sex-specific associations whenever possible.

To our knowledge, only one previous study by Wu et al. (2024) examined potential associations with continuous CMRS. Using NHANES (2003–2018) data with 13,782 adult participants, Wu et al. reported that a continuous CMRS was negatively associated with PFAS containing PFHxS, PFNA, pefluorodecanoic acid (PFDE), perfluoroundecanoic acid (PFUA) and MeFOSAA in their qgcomp, WQS and BKMR mixture analyses. Their single-pollutant models also found negative associations with PFHxS, MeFOSAA, PFDE, and PFUA [66]. In comparison, we observed effect estimates in the negative direction for CMRS among females, although the 95% CIs included the null. The limited literature on CMRS merits continued investigations to gain a more comprehensive understanding of the health implications associated with PFAS exposure.

### PFAS and dyslipidemia

Epidemiological evidence examined in recent umbrella review and systematic review concluded that PFAS was associated with dyslipidemia [8, 13]. However, the direction of the associations varied depending on lipids outcomes examined. Accordingly, PFAS was associated with higher total cholesterol and LDL-C [8, 13] but for TG and HDL-C, recent epidemiological studies reported diverging associations [63, 66–68]. For example, Borghese et al. (2025) examined 282 adult Canadian females who participated in a pregnancy cohort follow-up study (2018–2021) and reported no associations between PFAS mixture consisted of PFOA, PFOS, PFHxS, PFDA, PFNA, PFUnDA, and MeFOSAA and both TG and HDL-C using WQS regression and qgcomp. They also reported null results from their single-pollutant PFAS analyses [67]. Meanwhile, an NHANES age-stratified mixture study (2003–2018; *n* = 5850) by Lin et al. (2025) using also WQS regression and BKMR observed negative associations between PFAS mixture containing PFOA, PFOS, PFHxS, PFDA, PFNA and MeFOSAA and TG and low HDL-C among middle-aged adults (aged 40–59). Similarly, they also found negative associations between PFDA and TG among middle-aged adults and between PFHxS and PFDA and TG among older adults (aged ≥ 60). For HDL-C, however, they observed positive associations with PFHxS and PFDA among all adults and additionally PFOS among young adults (aged 20–39) and MeFOSAA among middle-aged adults [63]. Positive associations between PFOS and HDL-C were also reported by Maranhao Neto et al. (2022) who examined the individual effects of PFOA, PFOS, PFDA, and PFNA in 479 Czechs adults aged 25 to 89. However, they did not report associations between PFAS and TG [68].

The inconsistent associations in the epidemiological literature indicate further need to examine associations between PFAS and TG and HDL-C. Currently, the exact mechanisms underlying PFAS toxicity on lipid metabolism in humans are not fully understood but PFAS have been found to disrupt hepatic function and lipid metabolism via binding to nuclear receptors and cell membranes [12]. Different PFAS structures, such as chain lengths and terminal groups, may have different binding affinity for receptors and cell membranes [69, 70], which may explain the diverging associations reported among epidemiological studies that included different PFAS in their mixtures [12]. A deeper understanding of potential underlying molecular mechanisms may further explain inconsistencies in epidemiological findings.

### PFAS and central adiposity

Besides glucose metabolism and dyslipidemia, MetS also encompasses central adiposity, which is frequently assessed in epidemiological studies using WC [11]. Evidence for the association between PFAS and central adiposity is inconsistent [11]. Two recent studies reported associations with lower WC. One of which is by Maranhao Neto et al. (2022) who reported negative associations between WC and PFNA and PFDA among a sample of 479 adults living in the Czech Republic [68]. The other is by Lind et al. (2022), where among their sample of 502 50-year-old Swedish adults, also observed negative associations between WC and serum PFOS, PFOA, PFNA and PFDA levels, but only among females [71]. We also observed negative associations with WC among females, although the 95% CI included the null. However, these two studies, along with the ours, are limited in the use of cross-sectional designs which makes it challenging to infer directionality. Furthermore, the mechanism in which PFAS act to induce obesity is mostly unknown. One possible explanation for the female-specific negative associations observed between plasma PFAS concentrations and WC in a cross-sectional context could be the influence of parity. As such, pregnancy and breastfeeding are associated with both lower concentrations of some PFAS as well as higher central obesity via excessive gestational weight gain and post-partum weight retention [67, 72]. Future studies of associations between exposure to PFAS and central adiposity in females should account for reproductive history characteristics.

### PFAS and hypertension

We observed null associations between mixtures of plasma PFAS concentrations, and the sum of five PFAS, with measures of blood pressure. Although the associations were null, we observed a positive trend among males and a negative trend among females. Similarly in individual models, we found that PFOA was positively associated with DBP in males. The literature surrounding PFAS and BP is limited, with most studies showing positive associations with some PFAS [11]. A systematic review and meta-analysis by Yang et al. (2023) that included 14 single-pollutant studies on hypertension also reported positive associations but with PFOS, PFHxS and PFDA only [73]. However, two recent mixture studies reported opposite effects. Similarly to our qgcomp results, null association was reported by Lin et al. (2023) in their PFAS mixture analysis with hypertension using BKMR among 394 Chinese females recruited in 2012 and 2013 [74]. In contrast, Wu et al. (2024), using qgcomp, found that PFAS mixture is positively associated with hypertension, as well as DBP and SBP, with PFNA as the main contributor among 10,794 adult participants from the China National Human Biomonitoring program (2017–2018) [75]. A recent single-pollutant study by Li et al. (2024) also reported positive associations between hypertension and PFDA while PFOA, PFOS, PFHxS, and PFNA showed null associations [76]. Moreover, they found no evidence of effect modification by sex [76]. The sex difference we observed has previously been reported in the literature [77]. PFAS has been found to affect BP through regulatory pathways with different roles in males and females, such as those involving the renin-angiotensin system, sympathetic nervous activity, and endothelin-1 [78, 79]. There is also evidence that estrogen promotes vasodilation and thus protects pre-menopausal women from hypertension [78]. Further investigations are therefore needed to understand the mechanisms through which PFAS may be associated with blood pressure, and how they vary by sex.

### Strengths and limitations

Our study leveraged three cycles of nationally representative data to examine the associations between PFAS and MetS and cardiometabolic risk among adults living in Canada. In addition to MetS, we analyzed a continuous measure of cardiometabolic risk (i.e., CMRS) that retains information on the underlying distributions of the components of MetS, thereby reducing the potential for misclassification and measurement error as well as providing increased statistical power. Unlike previous studies that have relied on self-reported medication use to assign MetS status [55, 80, 81], CHMS participants were asked to bring all medications with them to their scheduled clinic visit where drug identification numbers were recorded by staff, thereby reducing the potential for misclassification and underreporting. Despite these strengths, our findings should be interpreted with caution due to the following limitations. First, although MetS is a recognized clinical construct, analyses based on multicomponent outcomes may obscure component-specific effects and mask opposing associations. Therefore, we also examined the individual components (i.e., CMRFs). Second, in constructing CMRS, there is no standard set of CMRFs or calculation method, and a single composite score may not account for all relevant CMRFs [82]. We combined various known CMRFs [83] and constructed CMRS by using the unweighted mean of the CMRF Z-scores, which assumed that each CMRF contributed equally to cardiometabolic risk. This approach has been validated for research that evaluates cardiometabolic risk in adults [84–86]. Third, medication status (whether individuals were taking anti-hypertensive, lipid-lowering or diabetic medication) was not considered in the construction of CMRS. However, results were similar in our sensitivity analysis excluding individuals treated with any medication for hypertension, hyperlipidemia and diabetes (Supplemental Tables S5). In previous studies, similar results and patterns of association were also observed regardless of medication status [27, 84, 87]. Furthermore, we were unable to address medication-related side effects. For example, medications for glucose control that induce weight loss may influence other CMRFs, such as WC, and could have biased our observed associations towards the null. Fourth, construction of MetS and CMRS typically includes fasting blood glucose measures but our blood glucose measurements were obtained from non-fasted blood samples. We therefore included HbA1c, a reliable clinical indicator of elevated blood glucose, to determine MetS status and create CMRS. Using non-fasting glucose measurements may produce inaccurate results due to the variability in blood concentrations based on recent food intake. We found that, in our population, the prevalence of MetS determined using HbA1c is slightly higher compared to using non-fasting GLU (HbA1c: total 27.1%, males 25.5% and females 28.5% vs. GLU: total 26.4%, males 24.8% and females 27.8%). Fifth, rather than using the original exposure unit, qgcomp transforms PFAS concentrations into quartiles which results in a loss of information of the real concentrations. Specifically, the qgcomp effect estimates reflect a joint increase of all PFAS by one quartile instead of a fixed increase on the absolute scale. This means that the exposure change is not uniform among PFAS chemicals and may represent much larger differences on the absolute scale for some PFAS than others. Sixth, our analysis involved multiple independent statistical models, each with an equal and independent risk of type I error. As a result, some findings may have occurred by chance. We therefore emphasized the direction, magnitude, precision, and consistency of the modelling results, together with evidence from prior toxicological and epidemiological literature, to guide our interpretation of the findings. Seventh, the cross-sectional design of the CHMS limits our ability to infer temporality and therefore causality. Although we observed potential detrimental effects on glucose metabolism, we cannot rule out reverse causality whereby changes in cardiometabolic health may have affected PFAS metabolism. Prospective studies are needed to better characterize the effects of mixtures of PFAS. Lastly, although we adjusted for a number of sociodemographic and lifestyle characteristics, the potential for unmeasured confounding cannot be ruled out. For example, we were unable to adjust for menstruation or menopausal status. The hormonal and physiological changes that occur during menopause can impact cardiometabolic health and cessation of menstruation leads to a reduction in the elimination rate of PFAS [88]. We were also unable to consider kidney function, including albuminuria, in our analysis. Given the role of the kidneys in PFAS elimination, unmeasured renal dysfunction may influence PFAS concentrations, potentially biasing the observed associations. Future analysis should consider adjusting for these potential confounders when possible.

## Conclusions

The etiology of cardiometabolic health is complex and our understanding continues to evolve. Our findings suggest that, among the adult population living in Canada, exposure to a mixture of PFAS may have a detrimental effect on glucose metabolism, but does not appear to be associated with MetS or overall cardiometabolic risk. Prospective investigations are needed to develop a more comprehensive understanding of the cardiometabolic health risks of exposure to PFAS.

## Supplementary Information

Supplementary Material 1.

## Data Availability

The Canadian Health Measures Survey data that support the findings of this study are available at https://www.statcan.gc.ca/en/statistical-programs/document/5071_D5_V2.

## Abbreviations

%: Percentage
%Δ: Percent difference
∑ 5PFAS: Total PFAS
µg/L: Microgram per litre
6:2 Cl-PFESA: 6:2 chlorinated polyfluoroalkyl ether sulfonic acid
BKMR: Bayesian kernel machine regression
BP: Blood pressure
CHMS: Canadian Health Measures Survey
CMRF: Cardiometabolic risk factors
CMRS: Cardiometabolic risk score
CVD: Cardiovascular diseases
DBP: Diastolic blood pressure
GLU: Glucose
GM: Geometric mean
HbA1c: Glycated hemoglobin
HDL-C: High-density lipoprotein cholesterol
HOMA-IR: Homeostatic Model Assessment of Insulin Resistance
KoNEHS: Korean National Environmental Health Survey
LOD: Limit of detection
MAP: Mean arteriole pressure
MeFOSAA: 2-(N-Methyl-perfluorooctane sulfonamido) acetic acid
MetS: Metabolic syndrome
mmHg: Millimeters of mercury
mmol/L: Millimole per litre
n: Sample size
NHANES: National Health and Nutrition Examination Survey
PFAS: Per- and polyfluoroalkyl substances
PFDA: Perfluorodecanoic acid
PFDE: Pefluorodecanoic acid
PFHxS: Perfluorohexane sulfonate
PFNA: Perfluorononanoic acid
PFOA: Perfluorooctanoic acid
PFOS: Perfluorooctane sulfonate
PFUA: Perfluoroundecanoic acid
PFUnDA: Perfluoroundecanoic acid
pmol/L: Picomole per litre
PR: Prevalence ratio
qgcomp: Quantile g-computation
SBP: Systolic blood pressure
SD: Standard deviation
T2D: Type 2 diabetes mellitus
TG: Triglycerides
WC: Waist circumference
WQS: Weighted quantile sum
