# Supplementary material for: Exposure to per- and polyfluoroalkyl substances and adult cardiometabolic health: a Canadian Health Measures Survey mixture analysis
Source: Environ Health. 2026 Feb 11;25:18. doi: 10.1186/s12940-026-01271-1 (PMC12998208; doi:10.1186/s12940-026-01271-1)
Supplement: Supplementary file 1 — Supplementary Material 1. [file 12940_2026_1271_MOESM1_ESM.docx]

**Title:** Exposure to per- and polyfluoroalkyl substances and adult cardiometabolic health: a Canadian Health Measures Survey mixture analysis

**Authors:**Janice M.Y. Hu, Michael M. Borghese, Annie St-Amand

**Affiliations:**Environmental Health Science and Research Bureau, Health Canada, Ottawa, ON, Canada

**Corresponding author:**Michael Borghese, PhD
Environmental Health Science and Research Bureau, Health Canada,
251 Sir Frederick Banting Way, Ottawa, ON, Canada, K1A 0K9;
Michael.Borghese@hc-sc.gc.ca

Table of Contents

Supplemental Tables and Figures

[**Figure S1.** Directed acyclic graph for the relation among PFAS exposures, cardiometabolic health indicators and various demographic characteristics. 3](#_Toc204334083)

[**Table S1.** Survey-weighted distributions of plasma PFAS concentrations (ug/L) among participants aged 20-79 in the Canadian Health Measures Survey (2009-2011, 2016-2019), stratified by sex. 4](#_Toc204334084)

[**Figure S2.** Pairwise Spearman’s correlation coefficients plot of plasma PFAS concentrations. 5](#_Toc204334085)

[**Table S2.** Prevalence ratios (PR) and 95% confidence intervals for associations between each 2-fold increase in plasma PFAS concentrations and metabolic syndrome (MetS) among participants aged 20-79 in the Canadian Health Measures Survey (2009-2011, 2016-2019), using survey-weighted modified Poisson regression. 7](#_Toc204334086)

[**Table S3.** Mean differences (Δ) and 95% confidence intervals for associations between each 2-fold increase in plasma PFAS concentrations and cardiometabolic risk score (CMRS) among participants aged 20-79 in the Canadian Health Measures Survey (2009-2011, 2016-2019), using survey-weighted linear regression. 7](#_Toc204334087)

[**Table S4.** Percent differences (%Δ) and 95% confidence intervals (95% CI) for associations between each 2-fold increase in plasma PFAS concentrations and each cardiometabolic risk factor (CMRF) among participants aged 20-79 in the Canadian Health Measures Survey (2009-2019), using survey-weighted linear regression 8](#_Toc204334088)

[**Table S5.** Sensitivity analysis of overall joint effects (Ψ) and 95% confidence intervals and weights for associations between a one-quartile increase in the PFAS mixture and cardiometabolic risk score (CMRS) among participants aged 20-79 in the Canadian Health Measures Survey (2009-2011, 2016-2019), while excluding participants who reported taking medications, using survey-weighted qgcomp regression 9](#_Toc204334089)


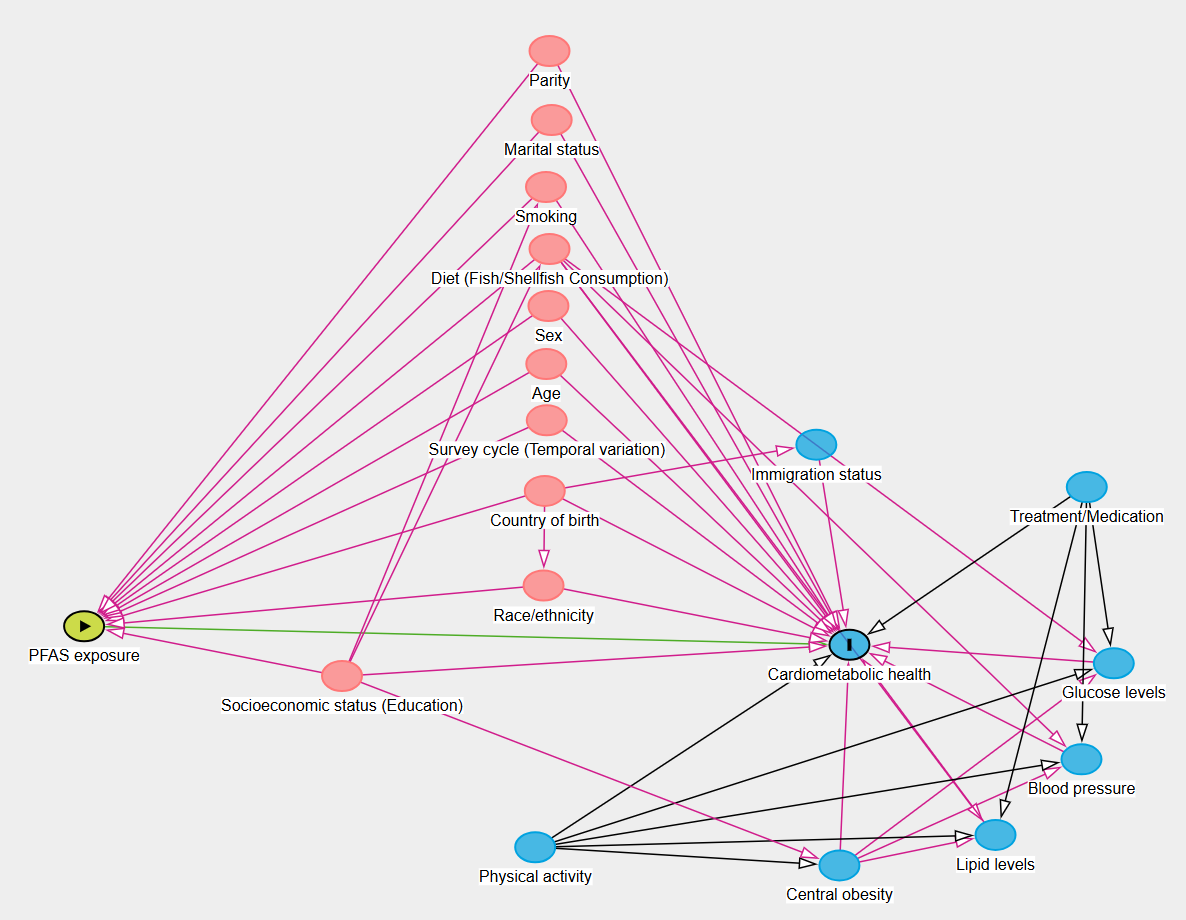


# **Figure S1.** Directed acyclic graph for the association among PFAS exposures, cardiometabolic health indicators and various demographic characteristics.

| **Table S1.** Survey-weighted distributions of plasma PFAS concentrations (ug/L) among participants aged 20-79 in the Canadian Health Measures Survey (2009-2011, 2016-2019), stratified by sex. | | | | | | | |
| --- | --- | --- | --- | --- | --- | --- | --- |
|  | **%<LOD** | **Percentiles** | | | | | **GM (95% CI)** |
|  |  | **5^th^** | **25^th^** | **50^th^** | **75^th^** | **95^th^** |  |
| **Total (n = 1071)** | | | | | | | |
| PFOA | 0 | 0.55 | 0.97 | 1.50 | 2.30 | 3.90 | 1.51 (1.39, 1.63) |
| PFOS | 0.1 | 1.00 | 2.40 | 3.80 | 6.70 | 17.0 | 3.98 (3.59, 4.41) |
| PFHxS | 0.5 | 0.23 | 0.62 | 1.10 | 1.90 | 5.60 | 1.08 (0.97, 1.21) |
| PFDA | 18.4 | <LOD | 0.11 | 0.20 | 0.28 | 0.63 | 0.19 (0.18, 0.21) |
| PFNA | 1.9 | 0.21 | 0.39 | 0.58 | 0.86 | 1.70 | 0.59 (0.54, 0.65) |
| ∑ 5PFAS | - | 2.63 | 4.93 | 7.88 | 12.46 | 27.98 | 7.91 (7.24, 8.63) |
| **Males (n = 513)** | | | | | | | |
| PFOA | 0 | 0.81 | 1.20 | 1.80 | 2.60 | 4.10 | 1.75 (1.59, 1.93) |
| PFOS | 0.1 | 1.50 | 3.30 | 4.90 | 7.80 | 17.00 | 5.18 (4.44, 6.05) |
| PFHxS | 0.5 | 0.56 | 0.99 | 1.50 | 2.60 | 7.30 | 1.63 (1.42, 1.88) |
| PFDA | 16.2 | <LOD | 0.11 | 0.20 | 0.29 | 0.59 | 0.19 (0.17, 0.21) |
| PFNA | 1.4 | 0.24 | 0.41 | 0.66 | 0.89 | 1.70 | 0.63 (0.57, 0.70) |
| ∑ 5PFAS | - | 3.75 | 6.33 | 10.10 | 13.76 | 28.50 | 9.99 (8.80, 11.34) |
| **Females (n = 558)** | | | | | | | |
| PFOA | 0 | 0.48 | 0.88 | 1.30 | 2.00 | 3.60 | 1.33 (1.20, 1.48) |
| PFOS | 0.1 | 0.82 | 1.80 | 3.10 | 5.50 | 15.00 | 3.19 (2.79, 3.64) |
| PFHxS | 0.5 | 0.20 | 0.40 | 0.85 | 1.30 | 3.40 | 0.77 (0.68, 0.87) |
| PFDA | 20.5 | <LOD | 0.11 | 0.20 | 0.27 | 0.69 | 0.19 (0.17, 0.22) |
| PFNA | 2.3 | 0.20 | 0.37 | 0.53 | 0.80 | 1.70 | 0.56 (0.49, 0.64) |
| ∑ 5PFAS | - | 1.98 | 3.90 | 6.11 | 10.30 | 27.38 | 6.49 (5.79, 7.27) |
| LOD – limit of detection; GM – geometric mean | | | | | | | |

1. Total


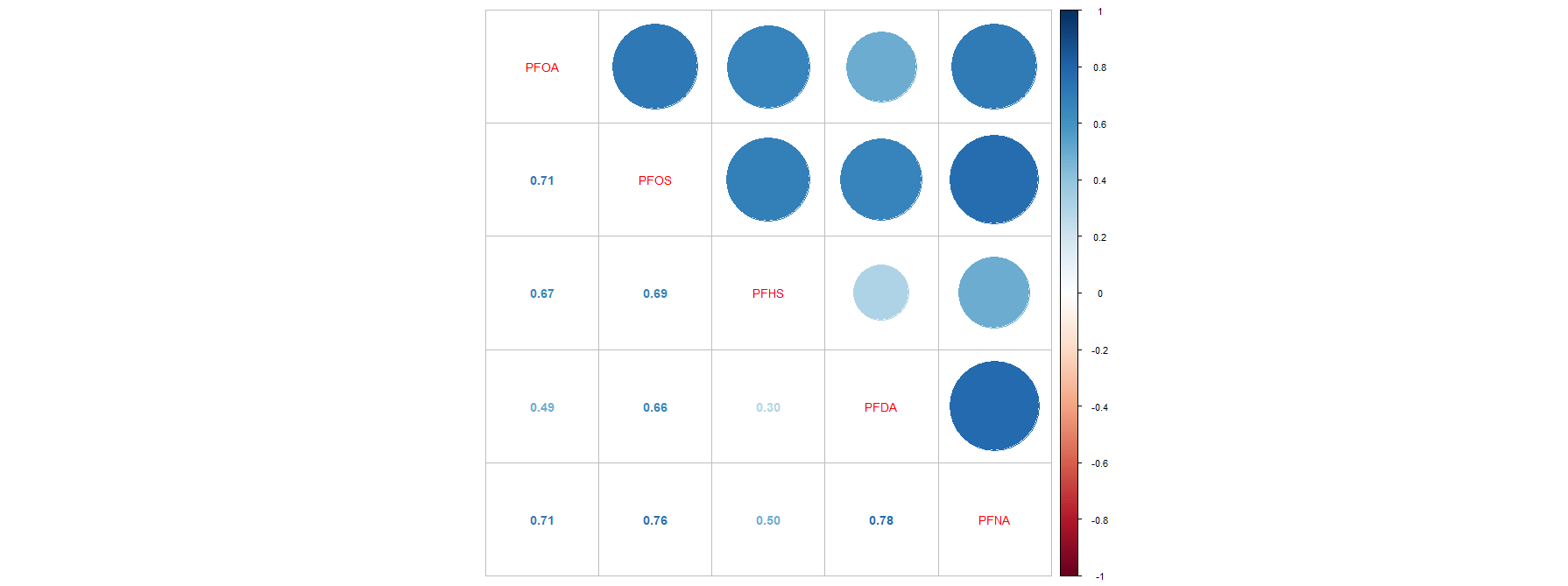


1. Males


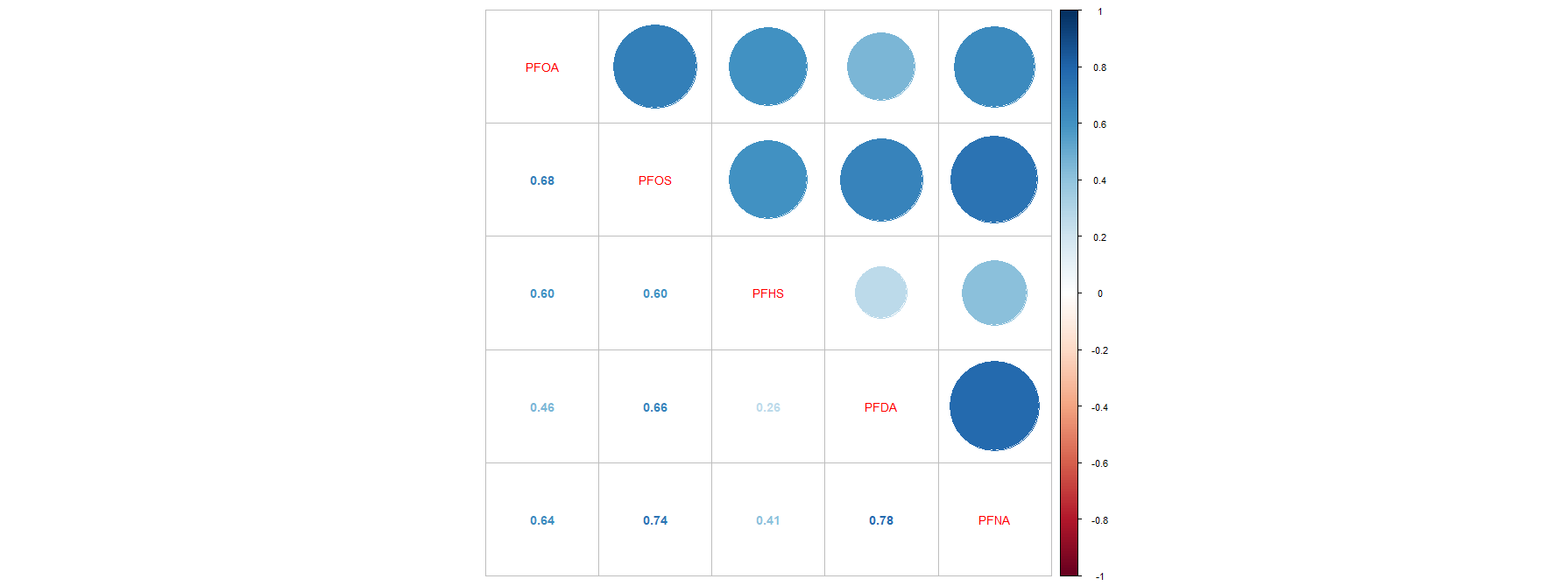


1. Females


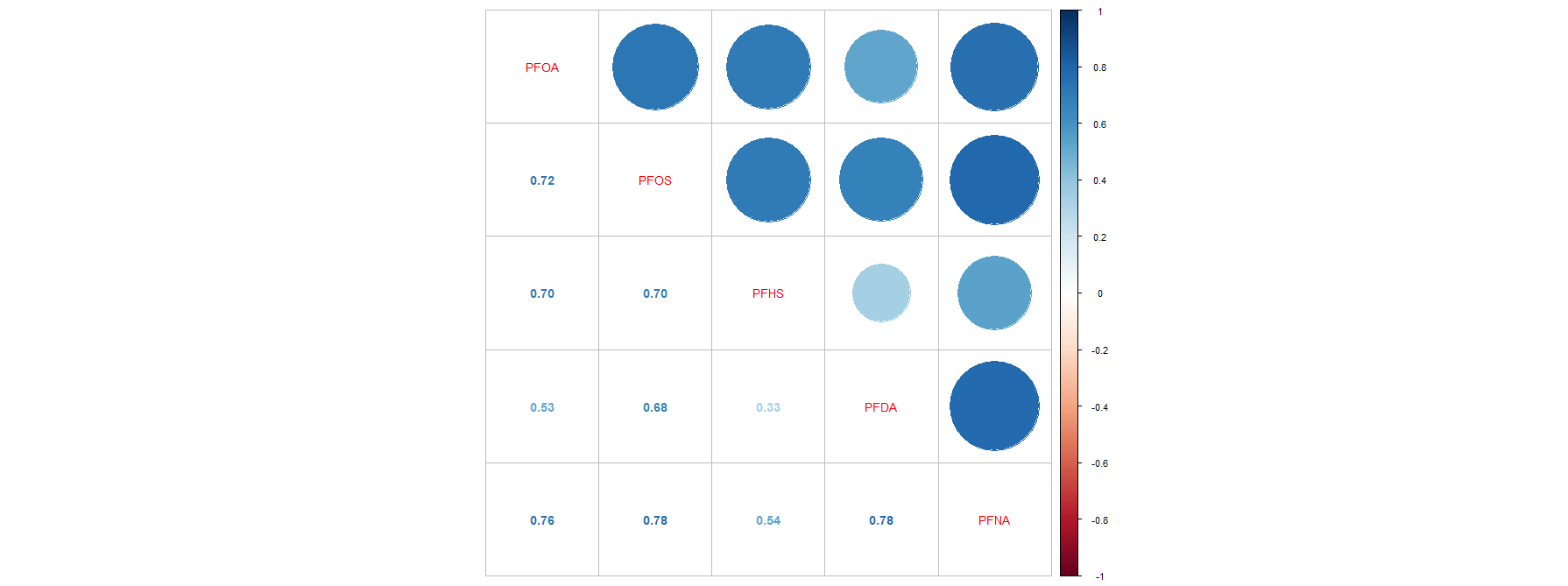


# **Figure S2.** Pairwise Spearman’s correlation coefficients plot of plasma PFAS concentrations.

| **Table S2.** Prevalence ratios (PR) and 95% confidence intervals for associations between each 2-fold increase in plasma PFAS concentrations and metabolic syndrome (MetS) among participants aged 20-79 in the Canadian Health Measures Survey (2009-2011, 2016-2019), using survey-weighted modified Poisson regression. | | | | | | |
| --- | --- | --- | --- | --- | --- | --- |
|  | **Total (n = 1071)** | | **Males (n = 513)** | | **Females (n = 552)** | |
|  | **Unadjusted**  **PR (95% CI)** | **Adjusted**  **PR (95% CI)** | **Unadjusted**  **PR (95% CI)** | **Adjusted**  **PR (95% CI)** | **Unadjusted**  **PR (95% CI)** | **Adjusted**  **PR (95% CI)** |
| PFOA | 1.18 (0.95, 1.47) | 0.99 (0.75, 1.32) | 1.35 (1.04, 1.76)* | 1.07 (0.82, 1.39) | 1.13 (0.84, 1.52) | 1.01 (0.64, 1.58) |
| PFOS | 1.24 (1.06, 1.45)* | 1.08 (0.92, 1.27) | 1.47 (1.20, 1.79)* | 1.06 (0.80, 1.39) | 1.19 (1.00, 1.43)* | 1.13 (0.92, 1.38) |
| PFHxS | 1.05 (0.92, 1.21) | 0.96 (0.85, 1.08) | 1.18 (1.02, 1.35)* | 1.07 (0.93, 1.25) | 1.04 (0.88, 1.24) | 0.93 (0.79, 1.08) |
| PFDA | 1.16 (0.97, 1.39) | 1.00 (0.80, 1.26) | 1.42 (1.14, 1.77)* | 0.98 (0.73, 1.31) | 1.04 (0.80, 1.35) | 1.03 (0.76, 1.37) |
| PFNA | 1.33 (1.16, 1.52)* | 1.16 (0.97, 1.38) | 1.45 (1.13, 1.86)* | 0.96 (0.71, 1.30) | 1.28 (0.94, 1.69) | 1.26 (0.94, 1.69) |
| ∑ 5PFAS | 1.26 (1.06, 1.51)* | 1.08 (0.90, 1.30) | 1.54 (1.23, 1.92)* | 1.09 (0.81, 1.47) | 1.21 (0.98, 1.51) | 1.12 (0.86, 1.45) |
| * p<0.05; All models are adjusted for age, race/ethnicity, education, marital status, smoking, country of birth, shellfish/fish consumption, physical activity and survey cycle.  Total models were additionally adjusted for sex and females models were additionally adjusted for parity. | | | | | | |

| **Table S3.** Mean differences (Δ) and 95% confidence intervals for associations between each 2-fold increase in plasma PFAS concentrations and cardiometabolic risk score (CMRS) among participants aged 20-79 in the Canadian Health Measures Survey (2009-2011, 2016-2019), using survey-weighted linear regression. | | | | | | |
| --- | --- | --- | --- | --- | --- | --- |
|  | **Total (n = 1071)** | | **Males (n = 513)** | | **Females (n = 552)** | |
|  | **Unadjusted**  **Δ (95% CI)** | **Adjusted**  **Δ (95% CI)** | **Unadjusted**  **Δ (95% CI)** | **Adjusted**  **Δ (95% CI)** | **Unadjusted**  **Δ (95% CI)** | **Adjusted**  **Δ (95% CI)** |
| PFOA | 0.04 (-0.20, 0.28) | 0.03 (-0.30, 0.35) | 0.21 (-0.01, 0.42) | 0.16 (-0.10, 0.42) | -0.01 (-0.43, 0.40) | -0.05 (-0.52, 0.43) |
| PFOS | -0.01 (-0.13, 0.11) | -0.07 (-0.21, 0.06) | 0.06 (-0.09, 0.21) | -0.10 (-0.30, 0.11) | -0.01 (-0.18, 0.16) | -0.08 (-0.24, 0.08) |
| PFHxS | -0.11 (-0.22, 0.08) | -0.13 (-0.26, -0.00)* | -0.07 (-0.25, 0.10) | -0.08 (-0.27, 0.10) | -0.11 (-0.27, 0.05) | -0.18 (-0.33, -0.03)* |
| PFDA | -0.01 (-0.21, 0.18) | -0.09 (-0.33, 0.15) | 0.08 (-0.14, 0.31) | -0.06 (-0.35, 0.22) | -0.08 (-0.36, 0.21) | -0.09 (-0.39, 0.22) |
| PFNA | 0.17 (-0.02, 0.35) | 0.14 (-0.13, 0.41) | 0.20 (0.01, 0.40)* | 0.07 (-0.19, 0.32) | 0.16 (-0.16, 0.48) | 0.15 (-0.24, 0.54) |
| ∑ 5PFAS | -0.02 (-0.17, 0.14) | -0.07 (-0.28, 0.14) | 0.04 (-0.13, 0.21) | -0.11 (-0.34, 0.13) | -0.00 (-0.23, 0.23) | -0.07 (-0.33, 0.18) |
| * p-value <0.05; All models are adjusted for race/ethnicity, education, marital status, smoking, country of birth, shellfish/fish consumption, physical activity and survey cycle.  Females models were additionally adjusted for parity. | | | | | | |

| **Table S4.** Percent differences (%Δ) and 95% confidence intervals (95% CI) for associations between each 2-fold increase in plasma PFAS concentrations and each cardiometabolic risk factor (CMRF) among participants aged 20-79 in the Canadian Health Measures Survey (2009-2019), using survey-weighted linear regression. | | | | | | |
| --- | --- | --- | --- | --- | --- | --- |
|  | **Total (n = 1071)** | **Males (n = 513)** | **Females (n = 552)** | **Total (n = 1071)** | **Males (n = 513)** | **Females (n = 552)** |
|  | **Adjusted**  **%Δ (95% CI)** | **Adjusted**  **%Δ (95% CI)** | **Adjusted**  **%Δ (95% CI)** | **Adjusted**  **%Δ (95% CI)** | **Adjusted**  **%Δ (95% CI)** | **Adjusted**  **%Δ (95% CI)** |
|  | **Waist circumference (WC)** | | | **Glucose (GLU)** | | |
| PFOA | 0.01 (-3.43, 3.56) | 2.16 (-0.25, 4.63) | -0.97 (-6.79, 5.21) | 1.57 (0.08, 3.08)* | 2.75 (0.50, 5.06)* | 1.07 (-1.27, 3.48) |
| PFOS | -0.78 (-2.40, 0.87) | -0.24 (-2.51, 2.09) | -1.23 (-3.23, 0.81) | 1.20 (0.11, 2.31)* | 0.74 (-1.12, 2.63) | 1.39 (0.01, 2.79)* |
| PFHxS | -2.09 (-3.55, -0.59)* | -1.05 (-3.35, 1.30) | -2.45 (-4.18, -0.69)* | 0.51 (-0.37, 1.40) | 1.22 (-0.50, 2.98) | -0.01 (-0.97, 0.95) |
| PFDA | -1.31 (-3.33, 0.76) | -0.22 (-2.77, 2.39) | -1.94 (-4.96, 1.19) | 1.37 (-0.17, 2.96) | 0.44 (-2.06, 3.02) | 1.95 (0.12, 3.82)* |
| PFNA | 0.92 (-1.88, 3.81) | 1.06 (-1.17, 3.34) | 0.54 (-3.80, 5.08) | 1.71 (0.40, 3.04)* | 1.56 (-0.28, 3.43) | 1.69 (-0.10, 3.51) |
| ∑ 5PFAS | -1.27 (-3.70, 1.22) | -0.54 (-3.23, 2.22) | -1.62 (-4.51, 1.36) | 1.69 (0.54, 2.89)* | 1.73 (-0.53, 4.04) | 1.63 (0.17, 3.12)* |
|  | **Triglycerides (TG)** | | | **Systolic blood pressure (SBP)** | | |
| PFOA | 6.69 (-4.58, 19.28) | 4.49 (-7.15, 17.58) | 6.13 (-7.28, 21.47) | 0.79 (-1.51, 3.14) | 2.31 (-0.53, 5.23) | -1.18 (-3.86, 1.59) |
| PFOS | 4.11 (-3.01, 11.75) | -1.10 (-8.98, 7.47) | 4.70 (-3.74, 13.89) | 0.49 (-1.74, 2.77) | 0.38 (-1.87, 2.68) | 0.17 (-2.13, 2.53) |
| PFHxS | -2.03 (-8.18, 4.53) | 2.37 (-6.53, 12.12) | -5.17 (-12.09, 2.30) | 0.12 (-1.42, 1.70) | 0.00 (-1.66, 1.68) | -0.19 (-2.16, 1.82) |
| PFDA | 1.60 (-7.68, 11.81) | -5.81 (-14.30, 3.53) | 4.88 (-6.42, 17.54) | 0.18 (-2.30, 2.73) | 1.58 (-1.26, 4.50) | -0.30 (-2.88, 2.34) |
| PFNA | 9.60 (0.47, 19.55)* | 0.52 (-6.78, 10.76) | 11.40 (-0.55, 24.78) | 1.02 (-1.65, 3.76) | 1.86 (-0.77, 4.56) | 0.20 (-2.71, 3.20) |
| ∑ 5PFAS | 5.04 (-4.66, 15.75) | 0.56 (-9.61, 11.88) | 5.47 (-5.47, 17.68) | 0.63 (-1.98, 3.31) | 0.85 (-1.77, 3.53) | -0.06 (-2.76, 2.70) |
|  | **Diastolic blood pressure (DBP)** | | | **High density lipoprotein cholesterol (HDL-C)** | | |
| PFOA | 0.19 (-2.17, 2.60) | 3.70 (0.62, 6.87)* | -2.53 (-5.94, 1.00) | 0.78 (-4.41, 6.25) | 0.25 (-5.92, 6.83) | 1.72 (-7.35, 11.67) |
| PFOS | -0.53 (-2.93, 1.94) | 0.69 (-1.49, 2.91) | -1.45 (-4.48, 1.69) | 2.12 (-0.68, 5.02) | 6.44 (2.17, 10.89)* | 1.61 (-2.12, 5.48) |
| PFHxS | -0.45 (-2.24, 1.37) | -0.30 (-2.46, 1.90) | -0.81 (-3.04, 1.47) | 3.66 (-0.19, 7.65) | 5.58 (-0.68, 12.24) | 3.88 (0.22, 7.66)* |
| PFDA | -0.75 (-3.32, 1.89) | 2.02 (-0.97, 5.10) | -2.06 (-5.85, 1.87) | 1.79 (-2.11, 5.84) | 2.61 (-2.10, 7.54) | 1.05 (-4.64, 7.07) |
| PFNA | 0.44 (-1.99, 2.95) | 2.82 (-0.07, 5.79) | -1.14 (-4.76, 2.63) | -1.84 (-5.96, 2.46) | -0.09 (-5.03, 5.11) | -2.35 (-8.88, 4.65) |
| ∑ 5PFAS | -0.58 (-3.47, 2.40) | 0.95 (-1.74, 3.71) | -1.79 (-5.37, 1.93) | 2.42 (-2.44, 7.53) | 8.29 (1.07, 16.02)* | 1.17 (-4.50, 7.18) |
|  | **Glycated hemoglobin (HbA1c)** | | |  | | |
| PFOA | 0.80 (-0.13, 1.74) | 0.56 (-0.70, 1.83) | 0.86 (-0.58, 2.33) |  |  |  |
| PFOS | 0.97 (0.22, 1.73)* | 0.40 (-0.77, 1.57) | 1.19 (0.24, 2.15)* |  |  |  |
| PFHxS | 0.34 (-0.23, 0.91) | 0.65 (-0.25, 1.54) | 0.05 (-0.69, 0.79) |  |  |  |
| PFDA | 0.55 (0.34, 1.44) | -0.64 (-2.19, 0.93) | 1.28 (0.16, 2.41)* |  |  |  |
| PFNA | 1.42 (0.47, 2.37)* | 0.49 (-0.84, 1.83) | 1.97 (0.63, 3.33)* |  |  |  |
| ∑ 5PFAS | 1.10 (0.25, 1.96)* | 0.63 (-0.63, 1.90) | 1.25 (0.13, 2.38)* |  |  |  |
| * p-value <0.05; TG & HDL models excluded individuals on lipid-lowering medication; GLU and HbA1c models excluded individuals on diabetes medication; SBP and DBP models excluded individuals on antihypertensive medication.  All models are adjusted for age, race/ethnicity, education, marital status, smoking, country of birth, shellfish/fish consumption, physical activity and survey cycle.  Total models were additionally adjusted for sex and females models were additionally adjusted for parity. | | | | | | |

| **Table S5:** Sensitivity analysis of overall joint effects (Ψ) and 95% confidence intervals and weights for associations between a one-quartile increase in the PFAS mixture and cardiometabolic risk score (CMRS) among participants aged 20-79 in the Canadian Health Measures Survey (2009-2011, 2016-2019), while excluding participants who reported taking medications, using survey-weighted qgcomp regression. | | | | | | | |
| --- | --- | --- | --- | --- | --- | --- | --- |
| **Population** | **Ψ (95% CI)** | **Weights** | | | | | |
|  |  | **Direction** | **PFOA** | **PFOS** | **PFHxS** | **PFDA** | **PFNA** |
| Total (n =741) | -0.13 (-0.35, 0.08) | Positive | 0.19 |  |  |  | 0.81 |
|  |  | Negative |  | 0.30 | 0.22 | 0.48 |  |
| Males (n = 350) | -0.09 (-0.33, 0.14) | Positive | 0.45 |  |  |  | 0.55 |
|  |  | Negative |  | 0.31 | 0.33 | 0.37 |  |
| Females (n = 388) | -0.25 (-0.50, 0.03) | Positive |  |  | 0.14 |  | 0.86 |
|  |  | Negative | 0.51 | 0.03 |  | 0.46 |  |
| * p-value <0.05; All models are adjusted for race/ethnicity, education, marital status, smoking, country of birth, shellfish/fish consumption, physical activity, and survey weight and excluded those taking lipid-lowering, antihypertensive & diabetes medications. Females model was additionally adjusted for parity.  Positive and negative weights correspond to the proportion of the effect in a particular direction and each sums to 1. | | | | | | | |
